# Effect of *Centella asiatica* ethanol extract on zebrafish larvae ( *Danio rerio*) insomnia model through inhibition of Orexin, ERK, Akt and p38

**DOI:** 10.12688/f1000research.141064.2

**Published:** 2024-07-18

**Authors:** Zamroni Afif, Mochammad Istiadjid Eddy Santoso, Nurdiana, Husnul Khotimah, Irawan Satriotomo, Shahdevi Nandar Kurniawan, Hidayat Sujuti, Dheka Sapti Iskandar, Annisatul Hakimah

**Affiliations:** 1 Department of Neurology, Faculty of Medicine, University of Brawijaya, Dr Saiful Anwar General Hospital, Malang, Indonesia; 2 Doctoral Program, Faculty of Medicine, Universitas Brawijaya, Malang, East Java, Indonesia; 3 Department of Pharmacology, Faculty of Medicine, Universitas Brawijaya, Malang, East Java, Indonesia; 4 Indonesian Neuroscience Institute, Jakarta, Indonesia; 5 Department of Ophthalmology, Faculty of Medicine, Universitas Brawijaya, Dr Saiful Anwar General Hospital Malang, Malang, East Java, Indonesia; 6 Master Program Biomedical Sciences, Faculty of Medicine, Universitas Brawijaya, Malang, East Java, Indonesia

**Keywords:** Insomnia, Centella asiatica, Orexin, ERK, p38, Akt.

## Abstract

*Background:* Insomnia is difficulty initiating or maintaining sleep for at least three nights a week or more and lasting for at least 3 months. One of the molecules that play a role in the circadian rhythm of arousal system is *hypocretin/orexin.* Orexin activates the p38-MAPK signaling pathway and increases phosphorylated ERK1/2 levels. *Centella asiatica* (CA) has a role in the signal work of the MAPK/ERK, Akt, and p38 path in many various diseases.

*Methods:* The research method used is true laboratory experimental. The research approach used was randomized control group post-test only. Zebrafish embryos aged 0-7 dpf were used in this study. The treatment group consisted of 5 groups: normal, insomnia, insomnia + 2.5 μg/mL CA, insomnia + 5 μg/mL CA, and insomnia + 10 μg/mL CA. The locomotor motion of zebrafish larvae was observed using Basler cameras on days five-, six- and seven-day post fertilization (dpf), then analyzed by using Western Blot method.

*Results:* The results proved that exposure to CA extract was able to reduce the expression of orexin (91963 ± 9129) and p38 (117425 ± 6398) as an arousal trigger in the sleep-wake cycle, with the most optimal concentration of CA 5 μg/mL. Exposure to CA extract was also able to reduce the expression of ERK (94795 ± 30830) and Akt (60113.5 ± 27833.5) with an optimum concentration of CA 2.5 μg/mL.

*Conclusion:* Exposure to CA extract was able to improve the sleep activity of zebrafish larvae insomnia model by extending the total inactivity time ( *cumulative duration*) and shortening the duration of first sleep ( *latency to first*) in light and dark phases through inhibition of orexin, ERK, p38, and Akt.

## Introduction

The 5th edition of *the Diagnostic and Statistical Manual of Mental Disorders* (DSM-5) and the third edition of *the International Classification of Sleep Disorders* (ICSD-3) define insomnia as difficulty initiating or maintaining sleep at least 3 nights a week or more and lasting at least 3 months. ^1^ The prevalence in Europe and Scandinavia shows an increasing prevalence. British study of more than 20,000 adults saw an increase in the average prevalence of insomnia over a 15-year period from 35% in 1993 to 38.6% in 2007, while the prevalence of insomnia in the United States was about 27%. ^1^^,^^2^

Based on gender, insomnia is more common in women than in men, which is 50% greater in women than men. Old age, low economic status, hyperarousal conditions, and the presence of psychiatric or medical comorbidities are risk factors associated with insomnia. ^1^ The latest concept of insomnia is the disintegration of molecules that play a role in alternation of waking and sleeping rhythms in the brain. One of the molecules that play a role in the circadian rhythm of arousal is hypocretin/orexin and histamine, while those that play a role in sleep, such as *γ-aminobutyric acid* (GABA), adenosine, serotonin, melatonin, and *prostaglandin D2* (PGD2). ^3^

**Figure 1.**
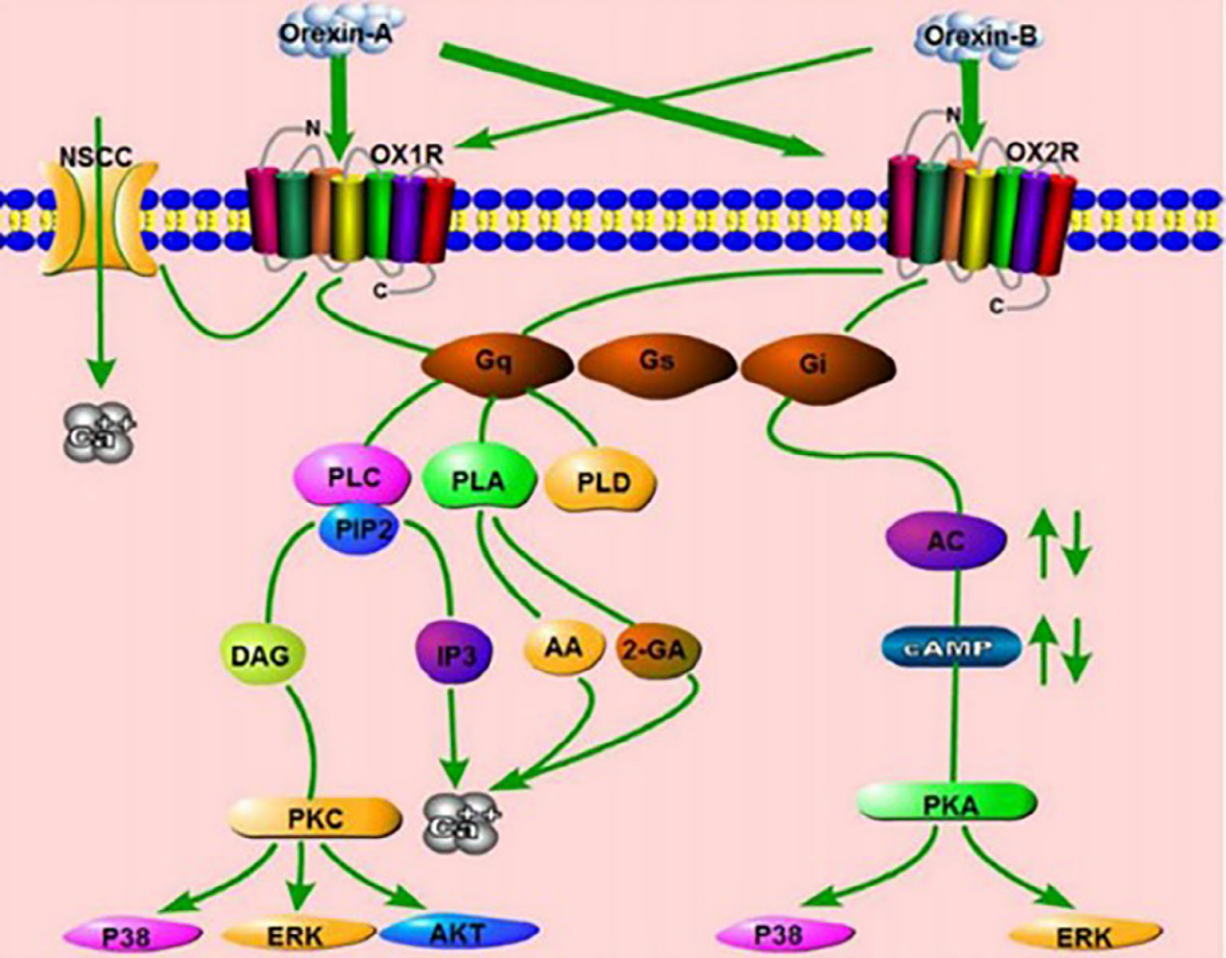
Orexin system signal pathway. ^3^

The binding of orexin to *orexin receptor type 1* (OXR1) or *orexin receptor type 2* (OXR2) stimulates the Gq or Gi subtypes, sequentially activating *phospholipase C* (PLC), *phospholipase A* (PLA), *phospholipase D* (PLD) or *Adenyl cyclase* (AC), causing an increase in cytosolic Ca ^2+^ and subsequent response cascades. OA binds to OX1R and increases Ca ^2+^ by activation of *nonselective cation channels* (NSCCs) ( Figure 1). ^3^

There are 2 receptor subtypes for orexin that will activate 3 G-protein subtypes (Gq/11, Gi/10, and Gs). Activation of Orexin Receptor-1 (OXR1 or OA) and Orexin Receptor-2 (OXR2 or OB) receptors activates *phospholipase c* cascade (PLC-IP3/DAG). Orexin receptor signaling pathways *phospholipase D* (PLD)/ *phosphatidic acid* (PA), *phospholipase A* (PLA)/ *arachidonic acid* (AA), and *mitogen-activated protein kinase* cascade (MAPK). Orexin activates the p38-MAPK signaling pathway and increases *phosphorylated* ERK1/2 levels. Extracellular Signal-regulated Kinases or ERK1/2 and p38 are rapidly phosphorylated in response to OA and OB, mediated primarily by Gq and slightly the Gi pathway. Orexin binding and orexin receptors will activate intracellular calcium *PKC-dependent* or independent pathways. Orexin receptors, both OA and OB, also stimulated the activation of *Akt kinase* in the cortical nerves of hypoxic stress-stressed rats. In addition, orexin will cause a reaction on OB to activate Gi proteins causing inhibition of cAMP formation. Orexin also activates OA to stimulate cAMP synthesis. Orexin signaling rapidly activates the mTORC1 pathway triggered by the *lysosomal v-ATPase* pathway. ^4^

*Centella asiatica* has a role in the signal work of the MAPK/ERK pathway. Studies on human breast cancer cells show that *Asiatic acid* (AA) in gotu kola plays a role in inducing inhibition of cancer cell growth through mediators such as MAPK, ERK1/2, and p38. *Asiatic acid* targets P13K/Akt/mTOR to prevent growth and induction of apoptosis in ovarian cancer cells. In cardiovascular disease AA has benefits with its ability to inhibit excessive pressure that induces cardiac hypertrophy by inhibition of phosphorylation of p38, MAPK, and ERK1/2 and decreases the production of TGF-β and NF-КB. ^5^^,^^6^

Currently, many animal models of sleep have been developed for sleep disorder research. The zebrafish ( *Danio rerio*) currently exhibit vertebral sleep models that are important among mouse and invertebrate models. Zebrafish sleep mainly at night as in humans, unlike rats that have the habit of sleeping during the day. The main advantages of zebrafish as sleep model animals are the discovery of genes involved in sleep control, neuropeptide systems, transparent anatomical structures of the brain, and allow for neuropsychiatric disease models, as well as models for genetic and pharmacological screening in sleep disorders. ^7^ Zebrafish brains also contain *orexin*, one of the molecules important for sleep regulation in mammalian brains. At present high-speed infrared video capture combined with computational image analysis has been used to quantitatively describe locomotor behavior and sleep posture in zebrafish larvae. ^3^ The purpose of this study was to prove the effect of gotu kola extract ( *Centella asiatica*) in improving the sleep activity of zebrafish larvae ( *Danio rerio*) insomnia model, extending the total inactivity time ( *cumulative duration*) and shortening the duration of first sleep ( *latency to first sleep*) in light and dark phases through inhibition of orexin, ERK, p38 and Akt.

## Methods

### Research design

The research design used in this study is *a true laboratory experiment.* The research approach used is randomized control group posttest only.

### Maintenance of zebrafish embryos

Maintenance of zebrafish embryos with exposure of dark:bright for 12:12 hours and plankton feeding are given three times a day, conductivity 350-600 S and salinity 0-0.6 ppt (parts per thousand). Zebrafish embryos aged three–seven- *day post fertilization* (dpf) was used in this study, obtained from the Reproductive Laboratory of the Faculty of Fisheries and Marine Sciences, Universitas Brawijaya.

This study used as many as three pieces of 48 well plates in each treatment group. The total number of embryos needed in this study was 144 embryos per group, so that the total number of zebrafish embryos included in the study in all treatment groups was 720 embryos. The Faculty of Medicine Health Research Ethics Committee Universitas Brawijaya has given its approval for this project. Ethics License No: 147/EC/KEPK-S3/06/2021.

The inclusion criteria in this study were healthy, clear embryos, not contaminated with fungi or parasites. Embryo hatching 2-3 dpf ( *day post fertilization*). While the exclusion criteria are zebrafish larvae that died or were deformed during the study.

The number of samples (n) in each treatment (p) is calculated based on the following formula with p = 7:

p(n-1) ≥ 15

pn-p ≥ 15

7n-7 ≥ 15

7n ≥ 22

n ≥ 4

From the calculations obtained n ≥ 4, to anticipate the possibility of illness and death in fish, the number of samples (n) used was 6 fish larvae in each group. After optimizing the sample in the protein isolation process before WB testing, it was found that the sample needed for each group was n = 144 zebrafish larvae, in this study there were 5 treatment groups, so that the total sample needed was 720 zebrafish larvae. This is because the type of sample in the form of larvae is very small (7 dpf) so it requires a large enough number of samples for protein isolation. However, as many as 6 larvae were recorded to be examined for locomotor motion in observing the cycle of wake and sleep larvae.

### Induction of insomnia

The light treatment used in this study to cause insomnia conditions in zebrafish larvae refers to the research method of Pinheiro-da-silva et al. (2016) with modification of light exposure as follows: negative control group with light exposure 12 hours light and 12 hours dark (normal group), and positive control group with light treatment 24 hours light (insomnia group). ^8^ Light is given at 200 lux from 0 dpf until the age of 7 dpf. Modifications were made to the design of lamps and tools used at the time of the study. ^9^

### Zebrafish sleep behavior analysis

Fish larvae movements were analyzed with video recordings using Ethovision XT software. The zebrafish larvae are sleep when the zebrafish is immobile for one minute. Sleep latency is the period when the lights are turned off and the first sleep period appears. Zebrafish larvae experience insomnia when the sleep latency is more than 20 minutes. ^9^

### Exposure to *Centella asiatica* extract

The administration of *Centella asiatica* ethanol extract with concentrations of 2.5 μg/ml, 5 μg/ml, and 10 μg/ml, sequentially introduced into the treatment group with 24 hours of light exposure group. *Centella asiatica* extract is given once in 24 hours at the same time as the media is replaced. The media referred to in this study, namely the solution used as a medium for maintaining zebrafish embryos. The chemicals needed in the manufacture of medium embryonic stock solution prepared in 1 liter contained 10 grams of NaCl, 0.30 grams of KCl, 1,630 grams of MgSO _4_, 0.4 grams of CaCl. ^9^

### Protein isolation

Zebrafish larva samples were lyzed in a suitable 5x RIPA buffer volume [50 mM Tris-Cl pH 7.4, 750 mM NaCl, 0.5% Sodium Deoxycholate, 0.5% SDS, 5% Triton, 1 mM Sodium Orthovanadate, 50 mM NaF, 5 μg/ml Pepstatin, 5 μg/ml Aprotinin, 5 μg/ml Leupeptin] on ice for 30 min followed by centrifugation at 14000 g for ten min. One volume of 100% (w/v) TCA was added to four sample volumes and incubated for ten min at 4°C. After centrifugation at 14000 rpm for five minutes, the pellets are removed and washed twice with cold acetone. The pellets are heated to 95°C for five-ten minutes and mixed in 50 μl urea 6 M in 50 mM bicarbonate. Protein content was assessed using a micro-BCA assay. Five μL of sample was added to the 96-well microplate followed by 100 μL of BCA reagent. The plates were incubated in the dark for 30 min at 37°C and absorbance was measured at 560 nm and protein concentration was determined from the BSA standard curve. ^10^

### Western blot and gel doc chemiluminescence examination

Each sample consisted of six zebrafish larvae aged seven days. All cell extracts were prepared in suspension in *ice-cold lysis buffer* (50 mM Tris-HCl, pH 7.4; 150 mM NaCl; 1% NP-40 and 0.5% sodium deoxycholate) with protease inhibitors. The protein sample was separated with *sodium dodecyl sulphate-polyacrylamide gel electrophoresis* and transferred to *the polyvinylidene difluoride* membrane. The membrane is blocked with 5% fat-free milk then the membrane was probed with primary antibodies overnight at 4°, followed by incubation with *HRP-conjugated* secondary antibodies for 1 hour. The blot is formed using an ECL kit according to the manufacturer’s instructions. Densitometric analysis was demonstrated using *QuantityOne software* (Bio-Rad). ^5^

### Statistical analysis

All data were analyzed using SPSS 25, a parametric test. Data that were normally distributed and homogeneous were analyzed using One-way ANOVA statistical analysis to determine the difference between the treatment groups provided. If the p-value < 0.05, it was considered statistically significant. Results expressed in mean ± SD.

## Results

### Average western blot analysis of orexin protein expression

**Figure 2.**
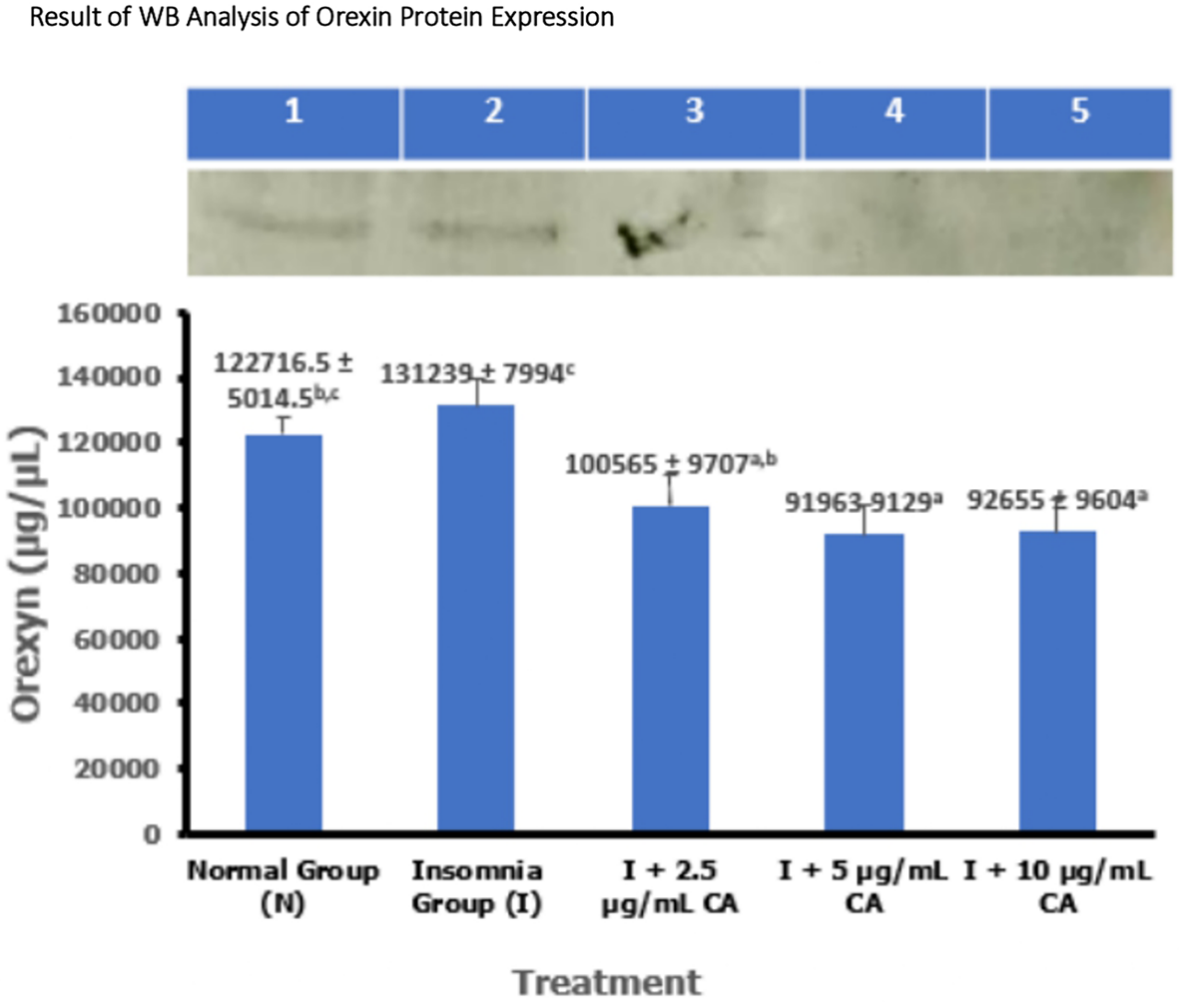
Diagram of the average result of WB analysis of orexin protein expression.

Protein expression of orexin was measured in zebrafish larvae samples using the western blot method and gel doc chemiluminescence. The average results of orexin protein WB analysis are presented in the form of tables and diagrams as follows ( Table 1 and Figure 2).

**Table 1.** Average Western blot analysis of orexin protein expression.

No.	Treatment	Number of samples (n)	Western blot protein expression of orexin ± SD (μg/μL)	p-value
1.	Normal Group (N)	144	122716.5 ± 5014.5 ^b,c^	<0.001
2.	Insomnia Group (I)	144	131239 ± 7994 ^c^	
3.	I + 2.5 μg/mL CA	144	100565 ± 9707 ^a,b^	
4.	I + 5 μg/mL CA	144	91963 ± 9129 ^a^	
5.	I + 10 μg/mL CA	144	92655 ± 9604 ^a^	

1. Normal Group (N) = kontrol negatif
2. Insomnia Group (I) = induksi cahaya
3. I + 2.5 μg/mL CA
4. I + 5 μg/mL CA
5. I + 10 μg/mL CA
6. Marker (Orexin)

Based on the average results of WB analysis of orexin protein expression, the insomnia group has a higher average expression value of 131239 ± 7994 μg/μL compared to the normal group (122716.5 ± 5014.5 μg/μL) and different concentrations of CA extract which are respectively 2.5 μg/mL (100565 ± 9707 μg/μL), 5 μg/mL (91963 ± 9129 μg/μL) and 10 μg/mL (92655 ± 9604 μg/μL). Various concentrations of CA extract can reduce the average expression value of orexin protein which basically functions as a wake-up trigger in the sleep-wake cycle. Statistical analysis also showed that there was a significant difference in orexin protein expression values, this was evidenced by significance signs in the form of ( ^a,b,c^) which showed that exposure treatment of CA extract was proven to reduce orexin protein expression values significantly lower than normal and insomnia groups. The location of orexin protein is at 49 kDa ( Table 1 and Figure 2).

### Average Western blot analysis of ERK protein expression

**Figure 3.**
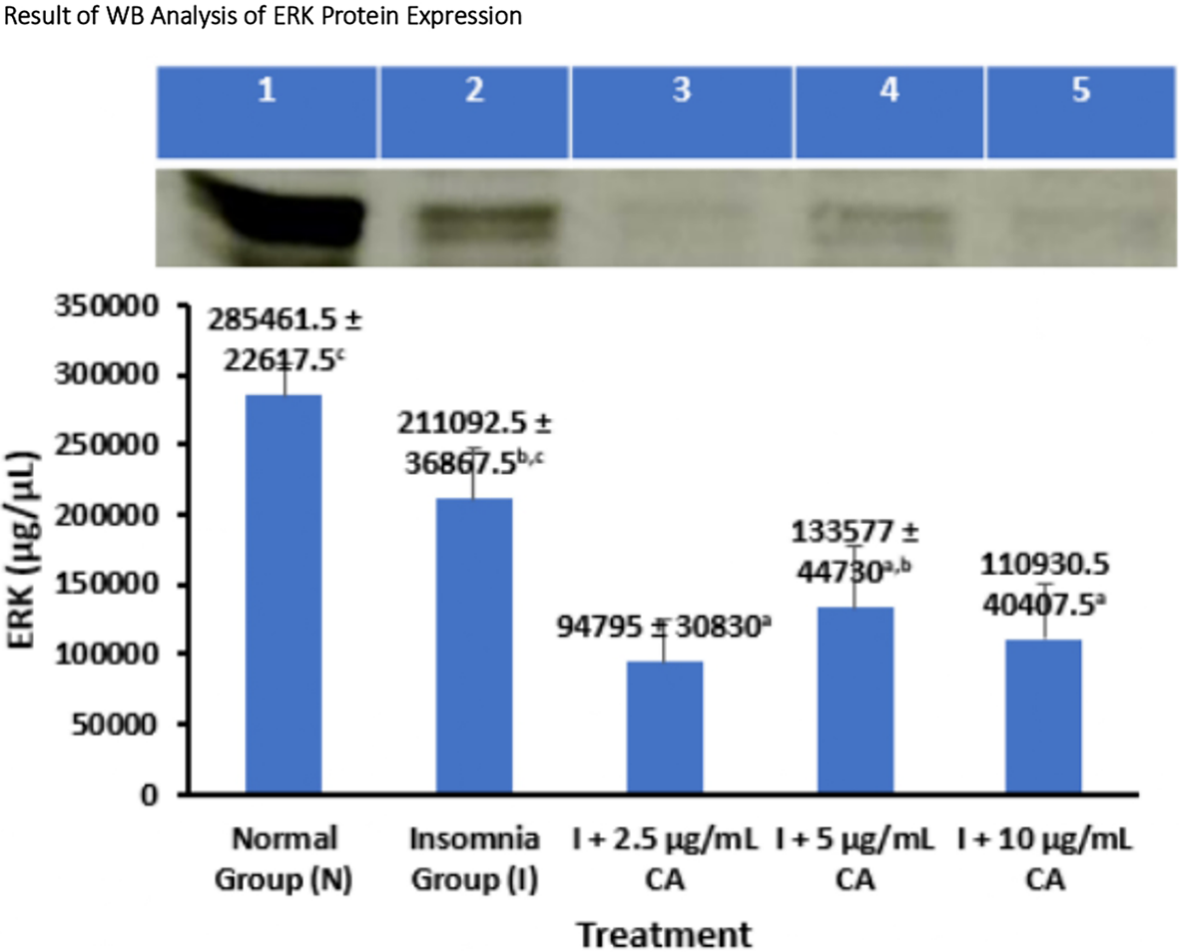
Diagram of the average result of WB analysis of ERK protein expression.

*Extracellular Signal Regulated Kinase* (ERK) protein expression was measured in zebrafish larvae samples using the western blot method and gel doc chemiluminescence. The average results of WB analysis of ERK protein are presented in the form of tables and diagrams as follows ( Table 2 and Figure 3).

**Table 2.** Average Western blot analysis of ERK protein expression.

No.	Treatment	Number of samples (n)	Western blot protein expression of ERK ± SD (μg/μL)	p-value
1.	Normal Group (N)	144	285461.5 ± 22617.5 ^c^	<0.001
2.	Insomnia Group (I)	144	211092.5 ± 36867.5 ^b,c^	
3.	I + 2.5 μg/mL CA	144	94795 ± 30830 ^a^	
4.	I + 5 μg/mL CA	144	133577 ± 44730 ^a,b^	
5.	I + 10 μg/mL CA	144	110930.5 ± 40407.5 ^a^	

Based on the average results of WB analysis of ERK protein expression, the normal and insomnia groups have higher average expression values of 285461.5 ± 22617.5 μg/μL and 211092.5 ± 36867.5 μg/μL compared to different CA extract concentration treatment groups, namely 2.5 μg/mL (94795 ± 30830 μg/μL), 5 μg/mL (133577 ± 44730 μg/μL), and 10 μg/mL (110930.5 ± 40407.5 μg/μL), respectively. Various concentrations of CA extract were able to reduce the average expression value of ERK protein which functions in the mechanism of orexin protein activation as a wake-up trigger in the sleep-wake cycle. Statistical analysis also showed that there was a significant difference in the value of ERK protein expression, this was evidenced by signs of significance in the form of ( ^a,b,c^) which showed that exposure treatment of CA extract was proven to reduce the value of ERK protein expression significantly lower than normal and insomnia groups. The location of the ERK protein is 42 kDa ( Table 2 and Figure 3).

### Average Western blot analysis of AKT protein expression

**Figure 4.**
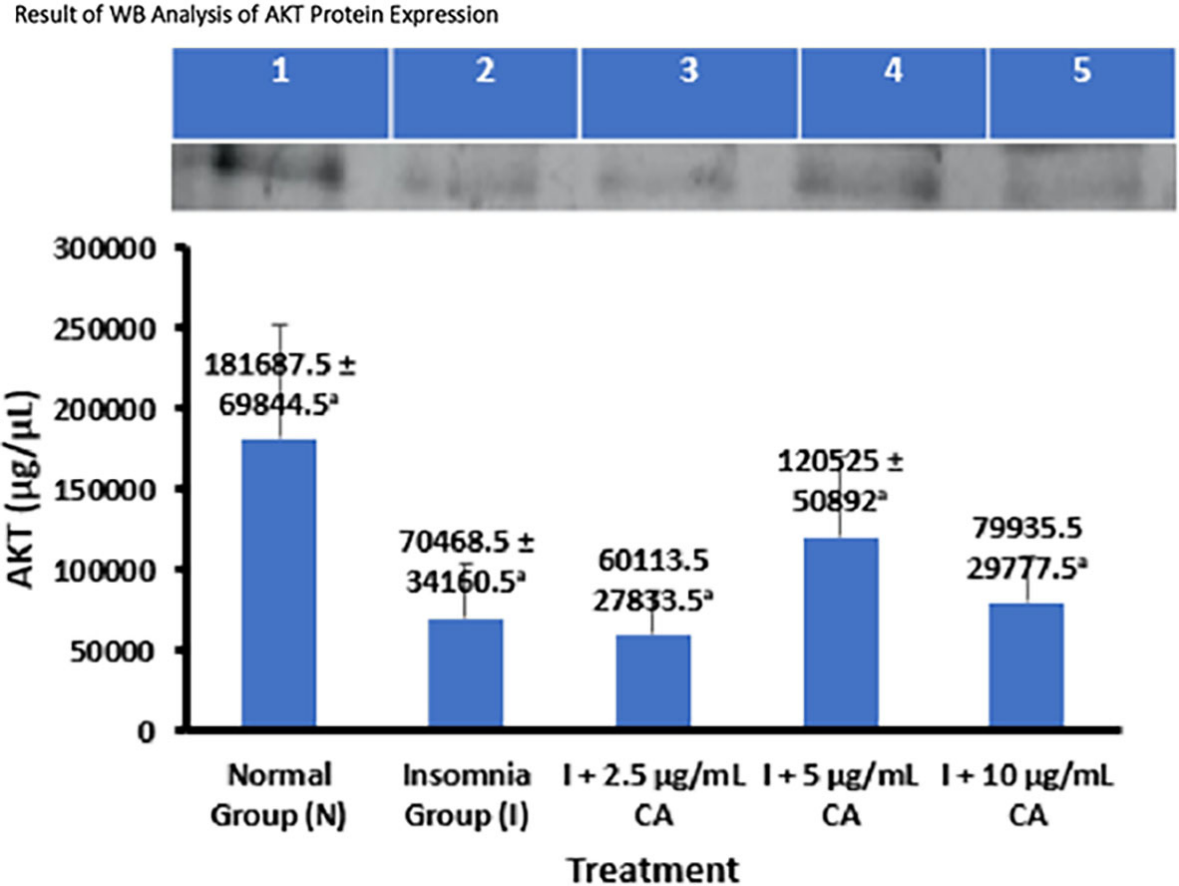
The average of WB analysis of AKT protein expression.

AKT Protein expression was measured in zebrafish larvae samples using western blot and gel Doc chemiluminescence methods. The average results of the AKT protein WB analysis are presented in the form of tables and diagrams as follows ( Table 3 and Figure 4).

**Table 3.** Average Western blot analysis of AKT protein expression.

No.	Treatment	Number of samples (n)	Western blot protein expression of AKT ± SD (μg/μL)	p-value
1.	Normal Group (N)	144	181687.5 ± 69844.5 ^a^	0.045
2.	Insomnia Group (I)	144	70468.5 ± 34160.5 ^a^	
3.	I + 2.5 μg/mL CA	144	60113.5 ± 27833.5 ^a^	
4.	I + 5 μg/mL CA	144	120525 ± 50892 ^a^	
5.	I + 10 μg/mL CA	144	79935.5 ± 29777.5 ^a^	

Based on the average results of WB analysis, Akt protein expression can be seen that the normal group and exposure treatment of CA extract concentration of 5 μg/mL have higher average expression values of 181687.5 ± 69844.5 μg/μL and 120525 ± 50892 μg/μL compared to the insomnia group (70468.5 ± 34160.5 μg/μL) and treatment of CA extract concentration of 2.5 μg/mL (60113.5 ± 27833.5 μg/μL) and 10 μg/mL (79935.5 ± 29777.5 μg/μL). The concentration of CA extract 2.5 μg/mL was able to reduce the average Akt protein expression value lower than the insomnia group. Statistical analysis shows that there is a difference in the value of Akt protein expression this is evidenced by the value of p-value = 0.045 (<0.05) which means there is a significant difference, but in post-hoc follow-up tests show that the sign of significance is in the form of ( ^a^) all which means that the average value of Akt expression for each group has no real difference. Therefore, from these results it can be concluded that the treatment of CA extract with the most optimal concentration of 2.5 μg/mL can improve sleep activity in zebrafish larvae insomnia models through decreased Akt protein expression. The location of the Akt protein is 60 kDa ( Table 3 and Figure 4).

### Average Western blot analysis of p38 protein expression

**Figure 5.**
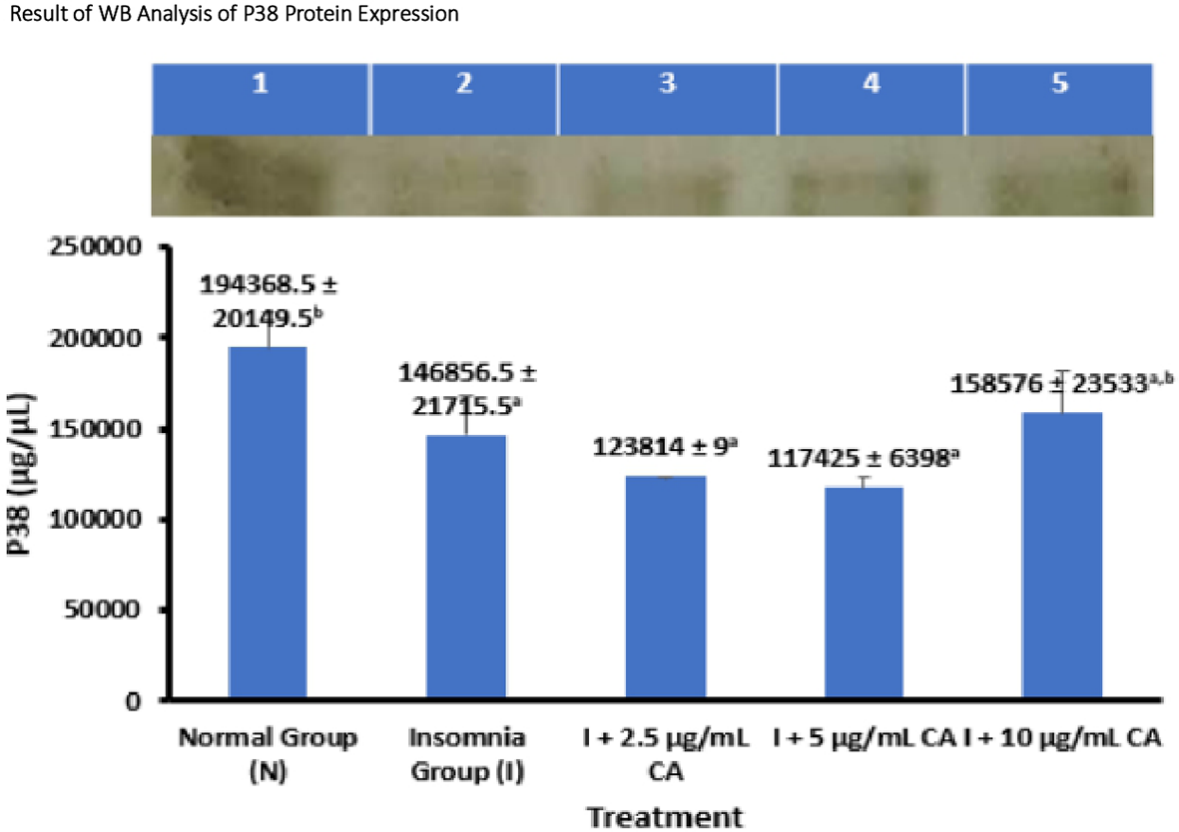
Diagram of the average result of WB analysis of p38 protein expression.

p38 Protein expression was measured in zebrafish larvae samples using western blot method and gel doc chemiluminescence. The average results of WB protein p38 analysis are presented in the form of tables and diagrams as follows ( Table 4 and Figure 5).

**Table 4.** Average western blot analysis of p38 protein expression.

No.	Treatment	Number of samples (n)	Western blot protein expression of P38 ± SD (μg/μL)	p-value
1.	Normal Group (N)	144	194368.5 ± 20149.5 ^b^	0.002
2.	Insomnia Group (I)	144	146856.5 ± 21715.5 ^a^	
3.	I + 2.5 μg/mL CA	144	123814 ± 9 ^a^	
4.	I + 5 μg/mL CA	144	117425 ± 6398 ^a^	
5.	I + 10 μg/mL CA	144	158576 ± 23533 ^a,b^	

Based on the average results of WB analysis of p38 protein expression, it can be seen that the normal group and the treatment of exposure to CA extract concentration of 10 μg/mL have a higher average expression value of 194368.5 ± 20149.5 μg/μL and 158576 ± 23533 μg/μL compared to the insomnia group (146856.5 ± 21715.5 μg/μL) and the treatment of CA extract concentration of 2.5 μg/mL (123814 ± 9 μg/μL) and 5 μg/mL (117425 ± 6398 μg/μL). The concentration of CA extract of 2.5 μg/mL and 5 μg/mL was able to reduce the average p38 protein expression value lower than the insomnia group. The p38 protein functions in the mechanism of orexin protein activation that can affect the sleep-wake cycle. Statistical analysis shows that there is a significant difference in the expression value of the p38 protein, this is evidenced by the value of p-value = 0.002 or <0.05 which means there is a significant difference. The location of the p38 protein is 38 kDa ( Table 4 and Figure 5).

### Average total inactivity time ( *cumulative duration*) in zebrafish larvae model of insomnia after exposure to *Centella asiatica* extract

a.
**Light phase**

**Figure 6.**
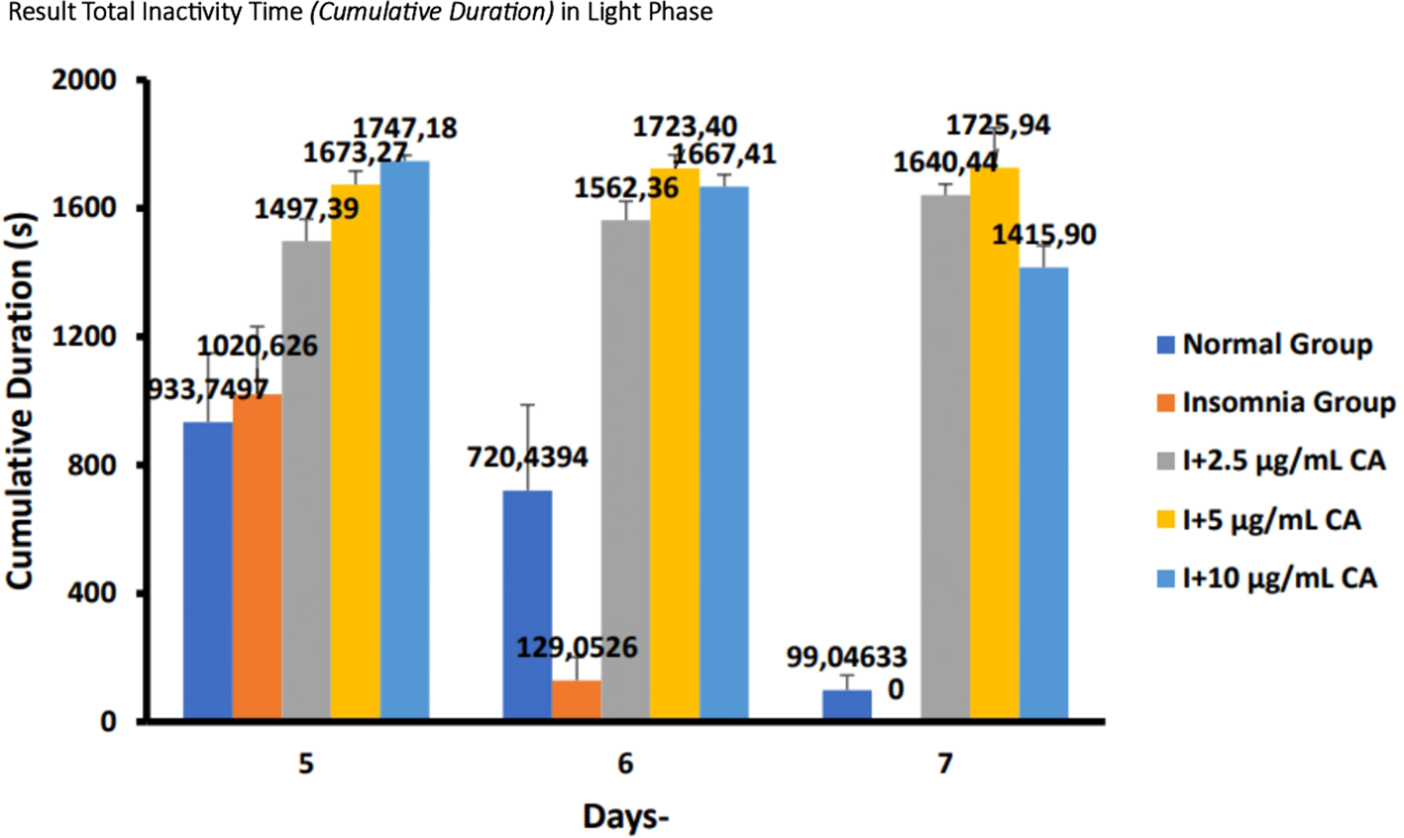
Diagram of the average result total inactivity time ( *cumulative duration*) in light phase.

The following are the results of fish motion analysis of total inactivity time ( *cumulative duration*) zebrafish larvae in the normal group, insomnia group (24-hour light exposure), and the group with the administration of CA extract concentrations of 2.5 μg/mL, 5 μg/mL, and 10 μg/mL during the light phase ( Table 5 and Figure 6).

**Table 5.** Average total inactivity time ( *cumulative duration*) of light phase.

No.	Treatment (n=6)	Days-Second (s)			p-value
		5	6	7	
1.	Normal Group (N)	933,7497 ^a^	720,4394 ^a^	99,04633 ^c^	<0.001
2.	Insomnia Group (I)	1020,626 ^a^	129,0596 ^c^	0 ^d^	
3.	I + 2.5 μg/mL CA	1497,39 ^b^	1562,36 ^b^	1640,44 ^b^	
4.	I + 5 μg/mL CA	1673,27 ^b^	1723,40 ^b^	1725,94 ^b^	
5.	I + 10 μg/mL CA	1747,18 ^b^	1667,41 ^b^	1415,90 ^b^

b.
**Dark phase**

**Figure 7.**
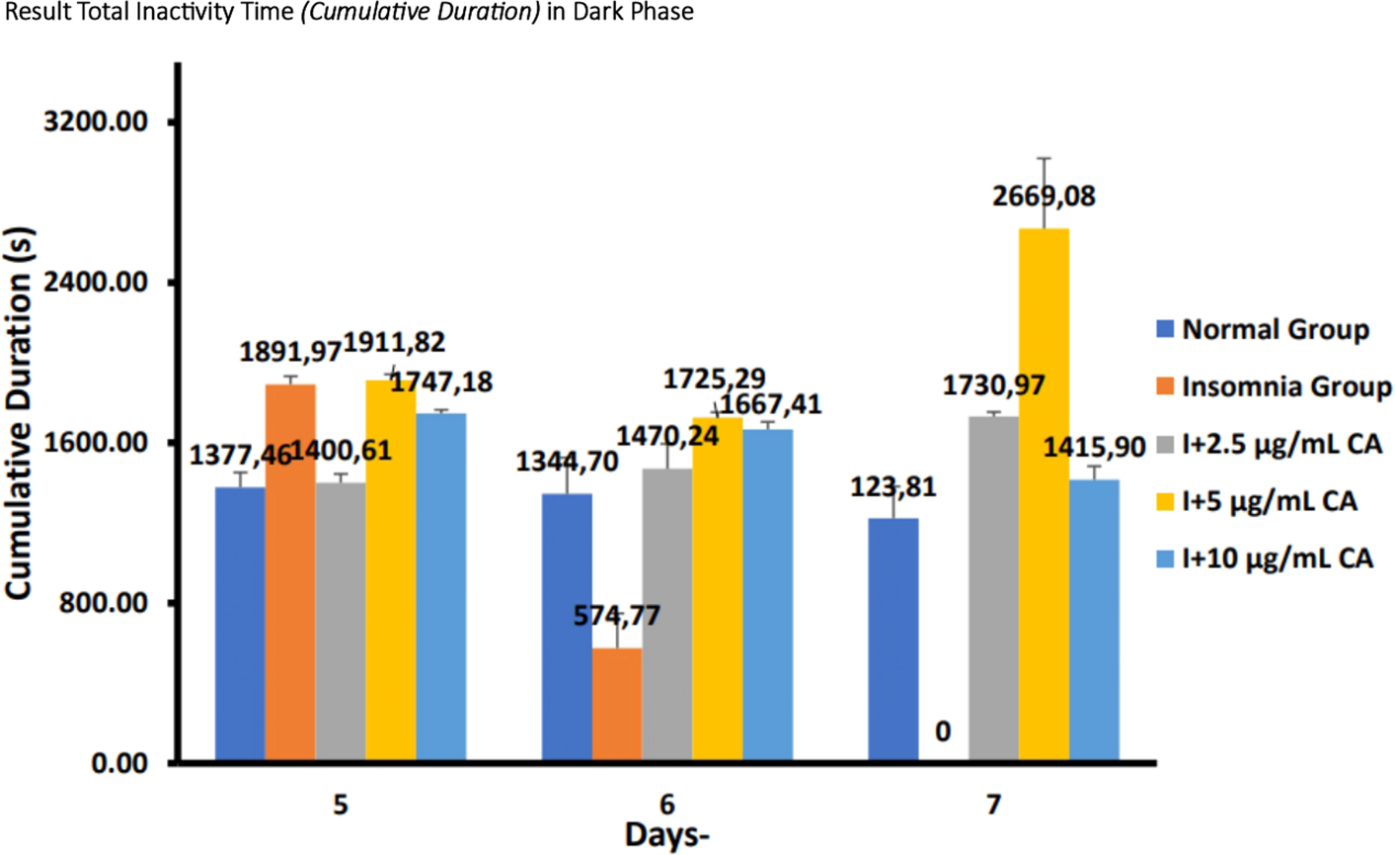
Diagram of the average result total inactivity time ( *cumulative duration*) in dark phase.

The following are the results of fish motion analysis of total inactivity time ( *cumulative duration*) zebrafish larvae normal group, insomnia group (24-hour light exposure), and group with CA extract concentrations of 2.5 μg/mL, 5 μg/mL, and 10 μg/mL during the dark phase ( Table 6 and Figure 7).

**Table 6.** Average total inactivity time ( *cumulative duration*) of dark phase.

No.	Treatment (n=6)	Days-Second (s)			p-value
		5	6	7	
1.	Normal Group (N)	1377,46 ^a^	1344,70 ^a^	1223,81 ^a^	<0.001
2.	Insomnia Group (I)	1891,97 ^b^	574,77 ^c^	0,00 ^d^	
3.	I + 2.5 μg/mL CA	1400,61 ^a^	1470,24 ^a^	1730,97 ^b^	
4.	I + 5 μg/mL CA	1911,82 ^b^	1725,29 ^b^	2669,08 ^b^	
5.	I + 10 μg/mL CA	1747,18 ^b^	1667,41 ^b^	1415,90 ^a^	

Based on the results of observations of locomotor motion of zebrafish larvae in normal groups, insomnia groups (24-hour light exposure), and groups with CA extract concentrations of 2.5 μg/mL, 5 μg/mL, and 10 μg/mL during light and dark phases, it can be concluded that exposure to CA extract with different concentrations can affect the total inactivity time ( *cumulative duration*) in zebrafish larvae to be longer than the insomnia group ( Tables 5, 6 and Figures 6, 7).

### Average total *latency to first sleep* in zebrafish larvae model of insomnia after exposure to *Centella asiatica* extract

**Figure 8.**
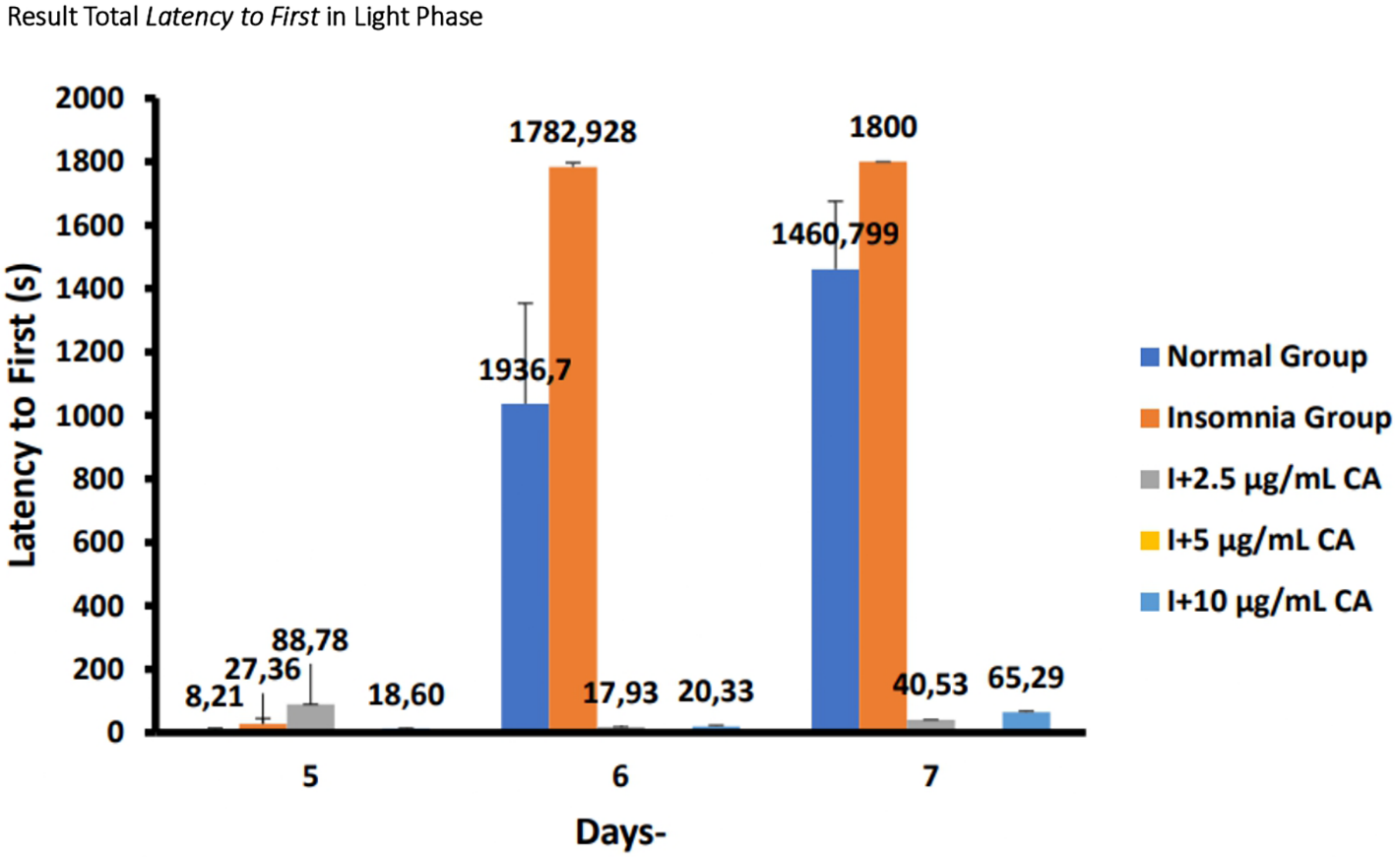
Diagram of the average result total *latency to first sleep* in light phase.

**Figure 9.**
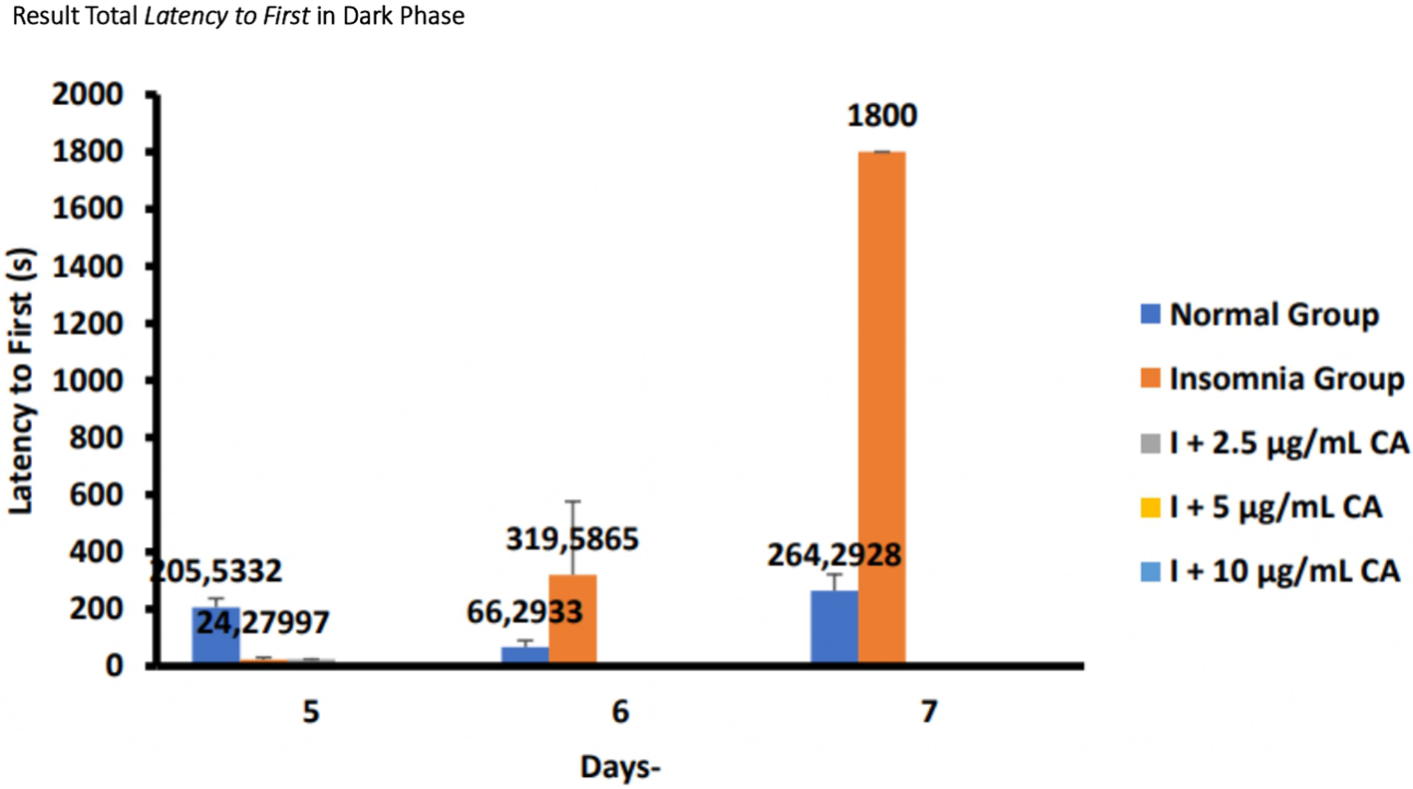
Diagram of the average total *latency to first sleep* in dark phase.

Based on the results of observations of locomotor motion of zebrafish larvae in normal groups, insomnia groups (24-hour light exposure), and groups with CA extract concentrations of 2.5 μg/mL, 5 μg/mL, and 10 μg/mL during light and dark phases, it can be concluded that exposure to CA extract with different concentrations can affect the average total time of first sleep onset ( *latency to first sleep*) in zebrafish larvae to be shorter than the insomnia group. This can be evidence that exposure to CA extract can improve insomnia conditions in zebrafish larvae by shortening the first time of sleep ( *latency to first sleep*) ( Tables 7, 8 and Figures 8, 9).

**Table 7.** Average total *latency to first sleep* light phase.

No.	Treatment (n=6)	Days-Second (s)			p-value
		5	6	7	
1.	Normal Group (N)	8,211105 ^a^	1036,7 ^d^	1460,799 ^d^	<0.001
2.	Insomnia Group (I)	27,3666 ^b^	1782,928 ^d^	1800 ^d^	
3.	I + 2.5 μg/mL CA	88,78 ^c^	17,93 ^a^	40,53 ^c^	
4.	I + 5 μg/mL CA	0,03 ^a^	0,03 ^a^	0,04 ^a^	
5.	I + 10 μg/mL CA	13,60 ^b^	20,33 ^a^	65,29 ^c^	

**Table 8.** Average total *latency to first sleep* dark phase.

No.	Treatment (n=6)	Days-Second(s)			p-value
		5	6	7	
1.	Normal Group (N)	205,5332 ^c^	66,2933 ^b^	264,2928 ^c^	<0.001
2.	Insomnia Group (I)	24,27997 ^b^	319,5865 ^c^	1800 ^d^	
3.	I + 2.5 μg/mL CA	24,37 ^b^	0,03 ^a^	0,03 ^a^	
4.	I + 5 μg/mL CA	0,29 ^a^	0,03 ^a^	0,04 ^a^	
5.	I + 10 μg/mL CA	0,04 ^a^	0,04 ^a^	0,04 ^a^	

## Discussion

Insomnia is primarily characterized by individual dissatisfaction with the duration or quality of sleep accompanied by functional impairment during the day. A person with insomnia usually experiences one or more symptoms such as fatigue, lack of energy, difficulty concentrating, and mood disorders. Insomnia can appear as a major complaint or more often occur along with other medical or psychiatric disorders, such as pain disorders and depression. ^11^ A 30-minute rule is used to meet insomnia criteria, including taking >30 minutes to fall asleep, spending >30 minutes awakening after sleep onset or waking >30 minutes before the desired time and before reaching 6.5 hours of sleep. ^12^

The latest concept of insomnia suggests the disintegration of molecules that play a role in alternation of wake and sleep rhythms in the brain. Molecules that play a role in the circadian rhythm of waking up are catecholamines, orexin, and histamine, while those that play a role for sleep, such as *γ-aminobutyric acid* (GABA), adenosine, serotonin, melatonin, and *prostaglandin D2* (PGD2). ^7^

*Hypocretin* (Hcrt) (also known as orexin) is a highly conserved *prepro-Hcrt* peptide product, with 2 enzymatically cleaved Hcrt peptides: Hcrt 1 or orexin A (33 amino acids) and Hcrt 2 or orexin B (28 amino acids). These peptides are synthesized in a group of neurons in the lateral *hypothalamus* (Hypo), and their neuronal processes extend widely in the brain. Mammals have 2 *Hcrt receptors* (HcrtRs) (HcrtR1/orexin A/OA receptor and HcrtR2/orexin B/OB receptor) that are distributed throughout the central nervous system and in peripheral organs. To date, a large amount of evidence suggests that Hcrt is involved in various physiological processes, such as sleep/wakefulness, food intake, and energy homeostasis. The HCRT system has also been reported to regulate reproduction. ^13^ Other studies have reported that in zebrafish, as in mammals, orexin signaling is involved in the regulation of many physiological functions, such as sleep/wake cycles, energy homeostasis, and locomotor activity. ^14^

*Hypocretin* (Hcrt) or orexin of the nearby hypothalamus synthesize and secrete Hcrts or orexin in the hypothalamus and are recognized primarily as important regulators of sleep or wakefulness, energy homeostasis, and appetite. Other researchers have previously shown that excessive orexin expression can cause insomnia-like phenotypes in zebrafish and that treatments such as orexin inhibition can stimulate feeding behavior in zebrafish. The effects of orexin on zebrafish sleeping and eating activity are very similar to what has been reported in mammals. ^13^

The main elements involved in the wake mechanism are orexin or hypocretinergic and histaminergic from the posterior portion of the lateral hypothalamus. Activated orexin will increase *phosphorylated ERK1/2* levels. ERK1/2 is rapidly phosphorylated in response to OA and OB, mediated primarily by Gq and slightly via Gi. In addition, dopamine and histamine cell groups have the highest density of *hypocretin-2 receptors* or OB. ^1^ Activated H1R ( *histamine-1 receptor*) will stimulate immune responses Th1 and Th2. Histamine H1 receptors are also expressed in skin dendrite cells and keratinocytes in skin tissue, and histamine increases NGF production over human keratinocyte H1R. NGF secretion is due to phosphorylation of protein kinase C, *extracelullar signal regulated kinase* (ERK), and activation of AP-1 as a result of H1 stimulation. ^15^

Another study explained that orexin, both OA and OB, also stimulated the activation of Akt kinase in the cortical nerves of hypoxic stress-stressed rats. In addition, Akt kinase also plays a role in several signaling pathways for apoptosis induction and ovarian cancer. ^3^ Orexin activates the p38-MAPK signaling pathway and increases *phosphorylated ERK1/2* levels. ERK1/2 and p38 are rapidly phosphorylated in response to OA and OB, mediated primarily by Gq and slightly through the Gi pathway. ^3^

*Centella asiatica* acts as a neuroprotector against various neurological disorders. The promising potential of *Centella asiatica* against neurological disorders can be attributed to its antioxidant, anti-inflammatory, anxiolytic, and stress-fighting properties. *Centella asiatica* in rats created with sleep deprivation for 72 hours significantly improved locomotor activity, anti-anxiety effects, lowered cortisol levels as well as improved neuronal inflammation, and apoptosis response. ^16^ *Centella asiatica* provides a calming effect on activity in experimental animals, can increase phenobarbitone which has the effect of inducing sleep time and reducing immobility in experimental animals. ^17^

Research by Kumar illustrates those 8 days of *Centella asiatica* treatment provides no benefit for inducing sleep but still shows a protective effect against sleep-induced anxiety ( *sleep deprivation*). The neuroprotective effects of *Centella asiatica* in sleep deprivation conditions that induce anxiety-like behaviors and the anxiolytic effects of *Centella asiatica* are modulated by NO ( *nitric oxide*). ^16^

Another study reported that one of the active compounds of *Centella asiatica* extract, *Asiatic Acid* (AA), showed an important role against *lipopolysaccharide* (LPS)/d-GaIN-induced fulminant hepatic failure with inhibition of oxidative stress and inflammation. The proposed mechanism is related to inhibition in MAPK and NF-КB. This study showed that AA induces anti-inflammatory effects through the activation of antioxidant enzymes by suppressing CCI and MAPK activity (p38, ERK1/2, and JNK, as well as increasing Nrf2 activity). ^4^

The effect of one of the active substances of *Centella asiatica*, namely *Asiatic acid* (AA) on P13 kinase (P13K)/Akt/mTOR has also been investigated as a therapeutic target in ovarian cancer cells. Phosphorylation of P13K, Akt, and mTOR decreased in *AA-treated cells*, indicating inactivation of the P13K/Akt/mTOR pathway by AA. Asiatic acid has a target at P13K/Akt/mTOR to prevent growth and induction of apoptosis in ovarian cancer cells. ^5^ Another study on the benefits of *Centella asiatica* against inflammatory reactions is mainly played by *Centella asiatica* compounds, showed that AA protects human bronchial cells against oxidative and inflammatory damage through mitochondrial stability, decreases ROS and PGE2 production, and suppresses the expression of NADPH oxidase proteins, COX-2, NF-КB p65, and p-p38. ^5^

*Cumulative duration* is the total length of time the fish is asleep or in an inactive condition. The characteristics of zebrafish when asleep are that adult zebrafish stop swimming for 6 seconds, and zebrafish larvae stop for 1 minute, do not move at the bottom or surface, and are less sensitive to external stimuli. Zebrafish larval activity is very minimal at night. ^18^ Zebrafish have cells that are responsive to light ^19^ and the duration of exposure to light received by fish leads to a decrease in melatonin production which produces several inputs that promote excessive wakefulness in the brain. ^19^

*Sleep latency* or *latency to first sleep* is defined as the time ( *both day and night*) needed to cause the first sleep. ^9^ In this study, it was found that the lengthening of latency to first sleep time or sleep latency in zebrafish larvae models exposed to 24-hour light. Continuous exposure to light can cause this condition, according to the theory that zebrafish have endogenously controlled circadian rhythm behaviors that can be affected by light. In another study obtained with a treatment of 14 hours:10 hours of light: dark, it was found that zebrafish larval activity occurred maximally in the phase of the lights turned on. From this study, the average sleep latency at light ranged from 24.8 to 59.2 minutes on the 6th and 7th day experiments with light exposure during the day. ^9^ The regulation of light to the sleep-wake cycle is related to the regulation of melatonin and the *hypocretin/orexin system* (Hcrtr). As much as 60% of these latency conditions decrease at night, which indicates the formation of hcrtr. ^19^

## Conclusions

Based on this study, it can be concluded that the effect of gotu kola extract ( *Centella asiatica*) in improving the sleep activity of zebrafish larvae ( *Danio rerio*) insomnia model by extending the total inactivity time ( *cumulative duration*) and shortening the duration of the first time of sleep ( *latency to first sleep*) in light and dark phases through inhibition of orexin, ERK, p38 and Akt.

## Data Availability

Figshare. data supporting research results Effect of *Centella asiatica* ethanol extract on zebrafish larvae ( *Danio rerio*) insomnia model through inhibition of Orexin, ERK, Akt and p38, DOI: 10.6084/m9.figshare.24078519. ^20^ The project contains the following basic data:
• Graph of exposure light and concentration of Centella asiatica extract. xlsx • Graph of WB marker Orexin, ERK, AKT and P38.xlsx • Results of Statistical Analysis of cumulative duration light and dark phase.docx • Results of Statistical Analysis of latency to first sleep light and dark phase.docx • Results of Statistical Analysis of WB AKT, ERK, Orexyn, and p38.docx •
Figure of Result Western Blot Marker Orexin, ERK, AKT and P38.docx Data is available under the terms of the Creative Commons Attribution 4.0 International (CC-BY 4.0) license. Figshare: The ARRIVE Essential 10: author checklist - Effect of *Centella asiatica* ethanol extract on zebrafish larvae ( *Danio rerio*) insomnia model through inhibition of Orexin, ERK, Akt and p38, DOI: 10.6084/m9.figshare.24078519. ^20^ https://figshare.com/articles/dataset/preview_ARRIVE_10_checklist_author/24079407 ^21^
• The ARRIVE Essential 10: author checklist.pdf Data are available under the terms of the Creative Commons Attribution 4.0 International license (CC-BY 4.0).
