# Supplementary material for: Effect of Centella asiatica ethanol extract on zebrafish larvae ( Danio rerio) insomnia model through inhibition of Orexin, ERK, Akt and p38
Source: F1000Res. 2024 Jul 18;13:107. Originally published 2024 Feb 16. [Version 2] doi: 10.12688/f1000research.141064.2 (PMC11135610; doi:10.12688/f1000research.141064.2)
Supplement: Peer review history

# Reviewer report: Reviewer response for version 2

**DOI:** 10.5256/f1000research.167944.r304662

**Published:** 2024-07-23

**Authors:** Rimawati Tedjasukmana

**Affiliations:** 1 Universitas Kristen Krida Wacana, Jakarta, Indonesia

This is a much better version of this article. My only comment is that Figure 1 has a lot of abbreviations that need to be explained

Is the work clearly and accurately presented and does it cite the current literature?

Partly

If applicable, is the statistical analysis and its interpretation appropriate?

Yes

Are all the source data underlying the results available to ensure full reproducibility?

Yes

Is the study design appropriate and is the work technically sound?

Yes

Are the conclusions drawn adequately supported by the results?

Yes

Are sufficient details of methods and analysis provided to allow replication by others?

Yes

Reviewer Expertise:

Sleep disorders

I confirm that I have read this submission and believe that I have an appropriate level of expertise to confirm that it is of an acceptable scientific standard.

# Reviewer report: Reviewer response for version 1

**DOI:** 10.5256/f1000research.154476.r254620

**Published:** 2024-05-28

**Authors:** Rimawati Tedjasukmana

**Affiliations:** 1 Universitas Kristen Krida Wacana, Jakarta, Indonesia

This article is scientifically sound, with good methods and valid results. However, parts of it are lost in translation because of the writing.

 A. Some paragraphs are confusing. For example:

 1. Paragraph 3 of Introduction is a very important paragraph, because it explained the reasons behind the authors' decision to choose the proteins tthat they investigated. This paragraph is difficult to follow, therefore it needs a complete rewrite and better still adding a diagram will be very enlightening.

 2. The whole discussion needs a rewrite, because the authors went off in a tangent in this part. It is better to stick to a few important points, i.e state the findings of the study and elaborate, compare the findings with results of other studies, analyse the discrepancies.

 B. Confusing use of phrases. For example

 1. Latency to first (page 10 paragraph 2). It is better suited to use the phrase 'latency to first sleep'

 2. Total time of first sleep (page 10 paragraph 2). The authors were trying to explain 'latency to first'. It is better to use 'time to first sleep onset'.

 C. Some uncommonly used abbreviations have no explanations. and sometimes the authors forgot to write the units after some numbers in the results part

Is the work clearly and accurately presented and does it cite the current literature?

Partly

If applicable, is the statistical analysis and its interpretation appropriate?

Yes

Are all the source data underlying the results available to ensure full reproducibility?

Yes

Is the study design appropriate and is the work technically sound?

Yes

Are the conclusions drawn adequately supported by the results?

Yes

Are sufficient details of methods and analysis provided to allow replication by others?

Yes

Reviewer Expertise:

Sleep disorders

I confirm that I have read this submission and believe that I have an appropriate level of expertise to confirm that it is of an acceptable scientific standard.

# Response

**Published:** 2024-06-04

**Authors:** Annisatul Hakimah

**Affiliations:** Neurology, Universitas Brawijaya, Malang, Indonesia

This article is scientifically sound, with good methods and valid results. However, parts of it are lost in translation because of the writing.

 A. Some paragraphs are confusing. For example:

 1. Paragraph 3 of Introduction is a very important paragraph, because it explained the reasons behind the authors' decision to choose the proteins tthat they investigated. This paragraph is difficult to follow, therefore it needs a complete rewrite and better still adding a diagram will be very enlightening.

**Answer:** we will list images related to the signal path of the Orexin system. We will send you pictures via email.

 2. The whole discussion needs a rewrite, because the authors went off in a tangent in this part. It is better to stick to a few important points, i.e state the findings of the study and elaborate, compare the findings with results of other studies, analyse the discrepancies.

 Answer: We have explained in the section related to important research findings that such as proteins used as markers, have proven their role in the mechanism of insomnia disease or circadian rhythm cycles (wake and sleep). And we have also compared the findings in this study which were later corroborated with other previous research theories to confirm the results of our study.

 B. Confusing use of phrases. For example

 1. Latency to first (page 10 paragraph 2). It is better suited to use the phrase 'latency to first sleep'

**Answer: **we will change the phrase "Latency to first" to "latency to first sleep".

 2. Total time of first sleep (page 10 paragraph 2). The authors were trying to explain 'latency to first'. It is better to use 'time to first sleep onset'.

**Answer: **we will change the phrase "Total time of first sleep" to "time to first sleep onset".

 C. Some uncommonly used abbreviations have no explanations. and sometimes the authors forgot to write the units after some numbers in the results part

**Answer:** because we have included units in the table and diagram of results, so we do not include in the explanation of results section, if it is necessary to include units in the explanation of results section, we will correct it.

# Reviewer report: Reviewer response for version 1

**DOI:** 10.5256/f1000research.154476.r254621

**Published:** 2024-04-24

**Authors:** Naricha Chirakalwasan

**Affiliations:** 1 Medicine, Chulalongkorn University, Bangkok, Bangkok, Thailand

Abstract - It is completed and summarized the rationale and the findings of the study.

 Introduction - It gave a good background on insomnia and underlying insomnia pathophysiology leading to this research project.

 Method-

1. Please clarify about how the authors calculated the number of embryos needed to be in the study. What was an alpha error and power that used for the calculation?
2. The study result was demonstrated as mean +/- SD, please confirm that the data has normal distribution and test of normality was conducted.

Result-

1. I did not see the data on the number of zebrafish in each group, please give this information in the text and tables, were they 144 in each group?
2. The meaning of superscript a,b, c should be clarified in the text and in each table so the readers understand what it is referring to and what group are being compared and whether the difference was statistically significant or not.
3. In Table 5-8, the cumulative duration (day-second (s)), should be explained since the data was shown as xxx,xxxx.
4. In table 5 and table 6, please explain why insomnia group had higher inactivity time (presumably sleep?) compared to normal group.
5. In table 6, giving the dose of 5 microgram/ml CA resulting in increase in inactivity time, however the dose of 2.5 and 10 microgram/ml CA resulting in reduction in inactivity time, please explain since if the CA is really working, it should result in dose dependent effect (increasing the dose associated with improving or increasing inactivity time).
6. In table 7 and 8, 5 microgram/ml CA resulting in latency to first light phase of 0 in all three days, however, some of the doses of 2.5 and 10 microgram/ml CA resulting in increase in latency to first light phase, please explain.

Discussion

1. First paragraphs are not relevant and suggested to be removed.
2. In the last paragraph (before the conclusion), “From this study, the average sleep latency at light ranged from 24.8 to 59.2 minutes on the 6th and 7th day experiments with light exposure during the day” this information does not match the data in table 7, please clarity.

Conclusion - It is concise.

Is the work clearly and accurately presented and does it cite the current literature?

Yes

If applicable, is the statistical analysis and its interpretation appropriate?

Partly

Are all the source data underlying the results available to ensure full reproducibility?

Yes

Is the study design appropriate and is the work technically sound?

Yes

Are the conclusions drawn adequately supported by the results?

Yes

Are sufficient details of methods and analysis provided to allow replication by others?

Partly

Reviewer Expertise:

Sleep Medicine, Pulmonary Medicine, Critical Care Medicine

I confirm that I have read this submission and believe that I have an appropriate level of expertise to confirm that it is of an acceptable scientific standard, however I have significant reservations, as outlined above.

# Response

**Published:** 2024-05-06

**Authors:** Annisatul Hakimah

**Affiliations:** Neurology, Universitas Brawijaya, Malang, Indonesia

Abstract - It is completed and summarized the rationale and the findings of the study.

 Introduction - It gave a good background on insomnia and underlying insomnia pathophysiology leading to this research project.

 Method-

 1. Please clarify about how the authors calculated the number of embryos needed to be in the study. What was an alpha error and power that used for the calculation?

**Response: **The number of samples (n) in each treatment (p) is calculated based on the following formula (Solimun, 2001) with p = 7:

 p(n-1) ≥15

 pn-p ≥ 15

 7n-7 ≥ 15

 7n ≥ 22

 n ≥ 4

 From the calculations obtained n ≥ 4, to anticipate the possibility of illness and death in fish, the number of samples (n) used was 6 fish larvae in each group. After optimizing the sample in the protein isolation process before WB testing, it was found that the sample needed for each group was n = 144 zebrafish larvae, in this study there were 5 treatment groups, so that the total sample needed was 720 zebrafish larvae. This is because the type of sample in the form of larvae is very small (7 dpf) so it requires a large enough number of samples for protein isolation. However, as many as 6 larvae were recorded to be examined for locomotor motion in observing the cycle of wake and sleep larvae.

 2. The study result was demonstrated as mean +/- SD, please confirm that the data has normal distribution and test of normality was conducted.

**Response: **All research data have been carried out statistical analysis of normality tests first before other tests and further tests. The data from the statistical analysis test has been listed in the Data availability point with DOI: 10.6084/m9.figshare.24078519. Data is available under the terms of the Creative Commons Attribution 4.0 International (CC-BY 4.0) license.

 Result-

 1. I did not see the data on the number of zebrafish in each group, please give this information in the text and tables, were they 144 in each group?

**Response: **The sample needed for each group is n = 144 zebrafish larvae, this study there are 5 treatment groups, so the total sample needed is 720 zebrafish larvae. For tables that have been corrected with an additional number of samples will be emailed separately.

 2. The meaning of superscript ^a,b,c ^should be clarified in the text and in each table so the readers understand what it is referring to and what group are being compared and whether the difference was statistically significant or not.

**Response: **Statistical analysis also showed that there was a significant difference in the value of orexin protein expression, this was evidenced by a sign of significance in the form of ( ^a, b, c^) which showed that exposure treatment to CA extract was proven to reduce the value of orexin protein expression significantly lower than normal and insomnia groups. The notations ^a, b, c^ indicate that there are significant differences between groups. The group with the notation ^a^ differs significantly from the group with the notation ^b^, as well as the group with the nitation ^b^ differs significantly from the notation ^c^, and vice versa the group with the notation ^a^ is also significantly different from the group with the notation ^c^, and so on.

 3. In Table 5-8, the cumulative duration (day-second (s)), should be explained since the data was shown as xxx,xxxx.

**Response: **We will fix this in table sections 5-8. However, in tables 5-8 on the journal website, cumulative duration data figures have been displayed. Please inform us about this.

 4. In table 5 and table 6, please explain why insomnia group had higher inactivity time (presumably sleep?) compared to normal group.

**Response: **Light has a very strong effect on suppressing sleep in zebrafish (Yokogawa et al., 2007). By the time zebrafish are given light exposure, zebrafish will not sleep at all. Light can suppress the sleep of zebrafish. Zebrafish are sensitive to light and light suppresses the production of melatonin, a hormone that stimulates sleep in zebrafish. Light strongly suppresses zebrafish sleep by up to 90% causing a decrease in sleep. The rate of sleep disturbance may be good but sleep disturbance with light may still cause nonspecific effects through sensory stimulation and changes in the circadian clock (Bringmann, 2019). Our results showed that on day 5 zebrafish larvae still need circadian rhythm adjustment compared to days 6 and 7.

 5. In table 6, giving the dose of 5 microgram/ml CA resulting in increase in inactivity time, however the dose of 2.5 and 10 microgram/ml CA resulting in reduction in inactivity time, please explain since if the CA is really working, it should result in dose dependent effect (increasing the dose associated with improving or increasing inactivity time).

**Response: **Based on the results of this study, it can be concluded that the increase or decrease that occurs in the measured parameters in this study is related to the concentration of *Centella asiatica* extract, classified as still safe for oral consumption. From this study it can be seen that the most optimal concentration in resulting in increased inactivity time is at a concentration of 5 micrograms / ml CA. However, further research is needed to ensure the safety of concentration and the most effective dose of *Centella asiatica* extract, especially in insomnia conditions and several protein molecules that play a role in sleep activity.

*Centella asiatica* has a role as a therapy for sleep disorders, one of which is by inhibiting histamine. Histamine itself is a neurotransmitter in the brain that plays a role in waking up. Histamine binding to histamine H3 receptors activates Gαi/o signaling causing MAPK activation. H3R activation is involved in sleep-wake cycles, recognition, regulation of homeostasis of energy levels, and neurotransmission (Thangam et al., 2018).

 6. In table 7 and 8, 5 microgram/ml CA resulting in latency to first light phase of 0 in all three days, however, some of the doses of 2.5 and 10 microgram/ml CA resulting in increase in latency to first light phase, please explain.

**Response: ** Based on the results of this study, it can be concluded that the increase or decrease that occurs in the measured parameters in this study is related to the concentration of *Centella asiatica* extract, classified as still safe for oral consumption. However, further research is needed to ensure the safety of concentration and the most effective dose of *Centella asiatica* extract, especially in insomnia conditions and several protein molecules that play a role in sleep activity.

 Discussion-

 1. First paragraphs are not relevant and suggested to be removed.

**Response: **We appreciate the reviewer's suggestion to remove the first paragraph in the discussion section, but our purpose in that paragraph is to provide an explanation regarding references to locomotor motion observation methods related to zebrafish wake and sleep cycles.

 2. In the last paragraph (before the conclusion), “From this study, the average sleep latency at light ranged from 24.8 to 59.2 minutes on the 6th and 7th day experiments with light exposure during the day” this information does not match the data in table 7, please clarity.

**Response: ** The explanation in this paragraph leads to reference literature of other journals that we refer to as support for the results of our research.

 Conclusion - It is concise.

 Reference-

 E. B. Thangam *et al.*, “The Role of Histamine and Histamine Receptors in Mast Cell-Mediated Allergy and Inflammation: The Hunt for New Therapeutic Targets,” *Front. Immunol.*, vol. 9, no. AUG, Aug. 2018, doi: 10.3389/FIMMU.2018.01873.

 H. Bringmann, “Genetic sleep deprivation: using sleep mutants to study sleep functions,” *EMBO Rep.*, vol. 20, no. 3, pp. 1–14, 2019, doi: 10.15252/embr.201846807.

 T. Yokogawa *et al.*, “Characterization of sleep in zebrafish and insomnia in hypocretin receptor mutants,” *PLoS Biol.*, vol. 5, no. 10, pp. 2379–2397, Oct. 2007, doi: 10.1371/JOURNAL.PBIO.0050277.

# Reviewer report: Reviewer response for version 1

**DOI:** 10.5256/f1000research.154476.r254619

**Published:** 2024-03-27

**Authors:** Desak Ketut Indrasari Utami

**Affiliations:** 1 Universitas Udayana, Bali, Indonesia

Full report:

 Comments for **abstract:**

 The research objective should also be stated in the abstract, so that it relates to the research results.

**Introduction:**

 The points made in the introduction are appropriate.

**Method:**

 The method is clear and has been approved by the ethical commission, but the reviewer does not understand the term media used, where in the manuscript it is stated that Centella asiatica extract is given once in 24 hours at the same time as the media is replaced. It is necessary to explain a little about this media.

**Results:**

 It is clear.

 No special comments.

**Discussion:**

 The first paragraph of the discussion is not really necessary to discuss this research, so the discussion is more focused on the research results that have been obtained according to the research objectives to prove the hypothesis.

**Comment on the answer to the question no. 1**

 The work is presented clearly and accurately, but some of the citations for this work are more than 5 years, there is even literature from year 2010.

Is the work clearly and accurately presented and does it cite the current literature?

Partly

If applicable, is the statistical analysis and its interpretation appropriate?

Yes

Are all the source data underlying the results available to ensure full reproducibility?

Yes

Is the study design appropriate and is the work technically sound?

Yes

Are the conclusions drawn adequately supported by the results?

Yes

Are sufficient details of methods and analysis provided to allow replication by others?

Yes

Reviewer Expertise:

Sleep disorders

I confirm that I have read this submission and believe that I have an appropriate level of expertise to confirm that it is of an acceptable scientific standard.

# Response

**Published:** 2024-03-28

**Authors:** Annisatul Hakimah

**Affiliations:** Neurology, Universitas Brawijaya, Malang, Indonesia

Comments for abstract:

 The research objective should also be stated in the abstract, so that it relates to the research results.

 Answer comments on the abstract section:

 We agreed to add a goal sentence to the abstract section "The purpose of this study was to prove the effect of gotu kola extract ( *Centella asiatica*) in improving the sleep activity of zebrafish larvae (Danio rerio) insomnia model, extending total inactivity time ( *cumulative duration*) and shortening the duration of first sleep ( *latency to first*) in light and dark phases through inhibition of orexin, ERK, p-38 and Akt".

 Method:

 The method is clear and has been approved by the ethical commission, but the reviewer does not understand the term media used, where in the manuscript it is stated that *Centella asiatica* extract is given once in 24 hours at the same time as the media is replaced. It is necessary to explain a little about this media.

 Answer comments in the method section:

 We agree to add an explanation related to the media referred to in this study, namely the solution used as a medium for maintaining zebrafish embryos. The chemicals needed in the manufacture of medium embryonic stock solution prepared in 1 liter contained 10 grams of NaCl, 0.30 grams of KCl, 1,630 grams of MgSO _4_, 0.4 grams of CaCl.

 Discussion:

 The first paragraph of the discussion is not really necessary to discuss this research, so the discussion is more focused on the research results that have been obtained according to the research objectives to prove the hypothesis.

 Answering comments in the discussion section:

 In our opinion, in the initial paragraph of the discussion section, it needs to be explained because it is related to the process of observing the locomotor motion of zebrafish used in this study, namely the duration of video taking for 30 minutes of the light phase (end) and 30 minutes of the dark phase (beginning), the process of changing time from wakefulness to sleep.

 Comment on the answer to the question no. 1

 The work is presented clearly and accurately, but some of the citations for this work are more than 5 years, there is even literature from year 2010.

 Answering the comment of question no.1:

 After we discussed the literature used in writing this article with several citations of works from the last 5 years because this study is the latest research on insomnia cases using zebrafish models, so for the latest literature we have not been able to find so far, but we can ensure that this work is presented clearly and accurately and by citing the latest literature in some related explanations.
